# First-line pazopanib in patients with metastatic epithelioid hemangioendothelioma: a retrospective single-center analysis

**DOI:** 10.1007/s00432-025-06208-8

**Published:** 2025-04-26

**Authors:** Anton Burkhard-Meier, Vera Valerie Rechenauer, Vindi Jurinovic, Markus Albertsmeier, Michael Hoberger, Hans Roland Dürr, Alexander Klein, Thomas Knösel, Wolfgang G. Kunz, Andreas Mock, Ada Pusztai, Michael Völkl, Michael von Bergwelt-Baildon, Lars H. Lindner, Dorit Di Gioia, Luc M. Berclaz

**Affiliations:** 1 Department of Medicine III, University Hospital, LMU Munich, Munich, Germany; 2 Bavarian Cancer Research Center (BZKF), Munich, Germany; 3 Institute for Medical Information Processing, Biometry, and Epidemiology, University Hospital, LMU Munich, Munich, Germany; 4 Department of General, Visceral and Transplantation Surgery, University Hospital, LMU Munich, Munich, Germany; 5 Orthopedic Oncology, Department of Orthopedics and Trauma Surgery, University Hospital, LMU Munich, Munich, Germany; 6 Institute of Pathology, LMU Munich, Munich, Germany; 7 Department of Radiology, University Hospital, LMU Munich, Munich, Germany; 8 German Cancer Consortium (DKTK), Partner Site Munich, Munich, Germany; 9 Department of Medicine III, LMU University Hospital, Marchioninistr. 15, 81377 Munich, Germany

**Keywords:** Epithelioid hemangioendothelioma, Rare cancers, Metastasis, Pazopanib, Targeted therapy

## Abstract

**Purpose:**

Epithelioid hemangioendothelioma (EHE) represents an ultra-rare, translocated vascular sarcoma with a heterogeneous course of disease. The optimal systemic treatment for patients with advanced EHE remains unclear. We sought to evaluate the value of pazopanib (PAZ) as a first-line treatment in metastatic EHE.

**Methods:**

Thirteen patients with metastatic EHE and PAZ as a first-line treatment at our institution between 2012 und 2023 were reviewed and analyzed with regard to clinical outcomes.

**Results:**

At a median follow-up of 51.4 months, the median progression-free survival (PFS) and overall survival (OS) were 35.1 and 53.8 months, respectively. In patients with documented prior tumor progression (*n* = 10), the median PFS and OS were 12.6 and 105 months, respectively. In patients with serosal effusion/ systemic symptoms (*n* = 4), the median PFS and OS were 6.1 and 10.3 months. The clinical benefit rate of the overall cohort was 62% with no complete or partial responses. Two of four patients experienced a reduction of symptoms (pain and ascites reduction/hemoptysis, respectively) under treatment with PAZ. Toxicity was mainly gastrointestinal and manageable with dose reductions. Permanent treatment interruption due to toxicity was necessary in one patient.

**Conclusion:**

This is the first study to systematically report survival outcomes for PAZ as a first-line treatment in patients with metastatic EHE. PAZ is active and safe in patients with metastatic EHE and may be considered as an alternative to sirolimus for specific patient subgroups. RECIST criteria should be questioned for evaluation of treatment response in EHE.

## Introduction

Epithelioid hemangioendothelioma (EHE) is an ultra-rare vascular soft tissue sarcoma (STS) with a prevalence of < 1/1.000.000. It is molecularly characterized by the gene fusion of WWTR1 (WW Domain Containing Transcription Regulator 1) and CAMTA1 (Calmodulin Binding Transcription Activator 1) or alternatively of YAP (Yes-associated Protein) and TFE3 (Transcription Factor E3)(Errani et al. 2011; Antonescu et al. 2013; Righi et al. 2020). The clinical behavior of EHE is highly variable, with 5-year survival rates ranging between 20% and 70%. In approximately 50% of cases, EHE is diagnosed with metastatic spread mainly affecting the liver, lungs, and bones (Lau et al. 2011; Sardaro et al. 2014; Angelo Dei Tos et al. 2021; Blay et al. 2023).

In asymptomatic patients with metastatic or unresectable EHE, a current international consensus paper recommends an active surveillance to avoid overtreatment. In case of progression or symptoms, a systemic therapy is indicated. However, EHE are typically refractory to chemotherapy used in STS and there are no active systemic therapies specifically approved for EHE (Frezza et al. 2021). Currently, the mTOR inhibitor sirolimus is suggested as the preferred first-line therapy based on a case series within the Italian Rare Cancer Network (Stacchiotti et al. 2021a, b).

Antitumor activity in EHE has also been seen with multi-targeted tyrosine kinase inhibitors (TKI) with a strong vascular endothelial growth factor receptor (VEGFR) inhibiting component, such as pazopanib (PAZ) and sorafenib (Chevreau et al. 2013; Kollár et al. 2017). PAZ has been approved by the *European Medicines Agency* (EMA) for the treatment of patients with non-adipocytic STS who have previously received chemotherapy, based on a significant PFS benefit in the placebo-controlled PALETTE trial(Van Der Graaf et al. 2012). In a retrospective study including patients with vascular sarcoma treated within the approval-relevant phase II/III trials and in real life practice at centers of the *European Organisation for Research and Treatment of Cancer* (EORTC), PAZ showed promising activity in the subgroup of EHE. Limited by the small patient number (*n* = 10) and a large range of survival, the median PFS and OS were both 26.3 months with an ORR of 20% (Kollár et al. 2017). Based on this study, its mechanism of action, as well as its approval and established use in STS, PAZ was utilized as a first-line treatment for EHE in our institution until 2023. To date, there are no studies on PAZ as a first-line therapy in patients with EHE. This study aimed to evaluate its efficacy and toxicity and to identify patients who benefit most from this regimen.

## Materials and methods

### Patient selection and treatment

Eligible patients (age ≥ 18 years) had histologically proven EHE and received PAZ as a first-line therapy at our institution. Histopathologic diagnosis was reviewed by a specialized sarcoma pathologist (TK). When available and not yet performed at initial diagnosis, EHE diagnosis was reconfirmed by immunohistochemistry (CAMTA1 positivity) and/or fluorescence in situ hybridization analysis for WWTR1 and/or TFE3 gene rearrangements according to the updated *World Health Organization* (WHO) classification (Sbaraglia et al. 2021). Clinical, pathological, and outcomes data were extracted from our prospectively maintained Sarcoma database. Dates of death were determined with the help of the Cancer Registry of Bavaria. After reimbursement approval of the respective health insurance company, PAZ was applied according to the product information with a daily target dose of 800 mg.

### Monitoring

A clinical examination including the presence of EHE-related symptoms was recorded at baseline and during treatment. Toxicity was measured according to *Common Terminology Criteria for Adverse Events* (CTCAE) v.5 (Cancer Institute 2017). Computed tomography (CT), magnetic resonance (MR) and/or positron emission tomography with 18-fluorodeoxyglucose (FDG-PET-CT) scans were performed at baseline and repeated every three months. The images were reviewed by a radiologist with subspeciality in oncological imaging (WGK). *Response evaluation criteria in solid tumors (*RECIST) v1.1 were used to evaluate efficacy of systemic therapy. The clinical benefit rate was determined based on RECIST criteria, including complete responses (CR), partial responses (PR), and stable diseases (SD) assessed at 6 months. Clinical progression, defined as the worsening of serosal effusion and tumor-related systemic symptoms without evidence of RECIST progressive disease (PD), was also reported according to Stacchiotti et al. (Stacchiotti et al. 2021b).

### Statistical analysis

OS and PFS were analyzed with Cox proportional hazards regression. PFS was calculated as the time from start of PAZ treatment to the first of either disease progression according to RECIST 1.1 or death of any cause. OS was measured from the start of PAZ treatment until death of any cause. PFS and OS were censored at the date of last follow-up. The results with a p-value of ≤ 0.05 were considered statistically significant. Statistical analysis was performed using R software version 4.0.3 (R Foundation for Statistical Computing, Vienna, Austria).

## Results

### Patient characteristics

The clinicopathologic characteristics of the patient group are summarized in Table 1.

**Fig. 1:**
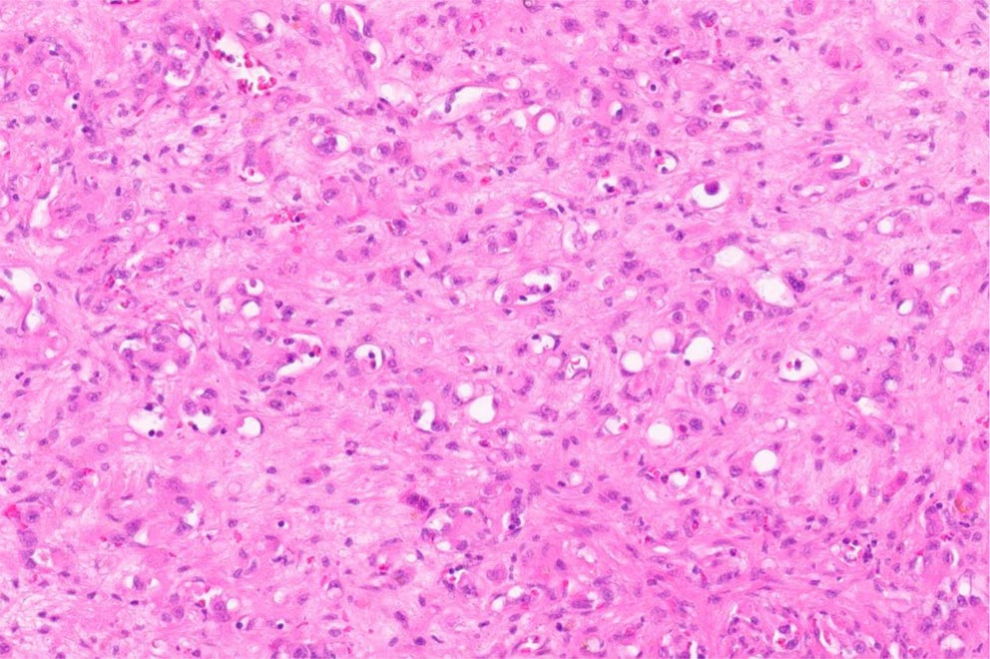
Epithelioid hemangioendothelioma with trabecular arrangement and nests of epithelioid cells within a sclerotic matrix (HE, 20x magnification)

**Fig. 2:**
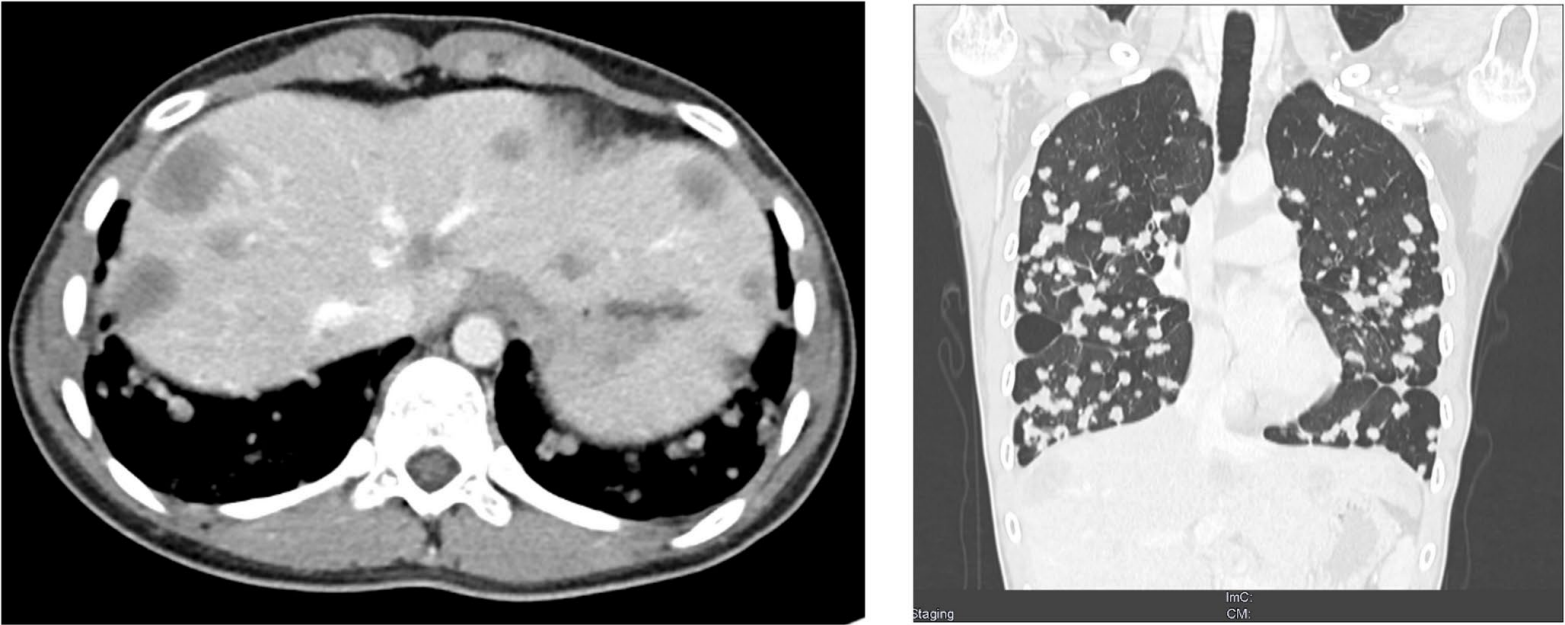
Baseline CT imaging of a patient with metastatic epithelioid hemangioendothelioma (EHE) involving liver and lungs

In total, 13 patients with metastatic EHE and start of PAZ therapy between December 2012 and February 2023 were included in this study. In ten patients (77%), a WWTR1::CAMTA1 fusion was confirmed by immunohistochemical or molecular assessment. In three patients (23%), EHE was initially diagnosed without confirmation of the characteristic gene fusion, and no material was available for re-review. Histomorphological features of EHE and a representative baseline CT imaging of a patient with metastatic EHE are illustrated in Figs. 1 and 2, respectively.

The median age was 43 years (range 22–68 years), and six patients (46%) were female. Twelve patients (92%) did not receive a prior systemic therapy, while one patient (8%) had been pretreated with gemcitabine/cisplatin due to the initial false diagnosis of a biliary tract cancer (see patient 11 in Fig. 5). One patient (8%) was treated with PAZ as part of the EPAZ trial (Grünwald et al. 2020). Eight patients (62%) presented with PD according to RECIST at start of PAZ, while two patients (15%) experienced only clinical progression characterized by symptoms and recurrent serosal effusions. Three patients (23%) showed no tumor progression; in these cases, the multidisciplinary tumor board recommended PAZ as a bridging therapy prior to a potential liver transplant. Four patients (31%) presented with symptomatic disease and serosal effusion at the initiation of treatment with PAZ. The median interval from initial diagnosis to the start of PAZ therapy was 105 days (range 43-1625 days). The median duration required for therapy approval by health insurance was 32 days (range 8-260 days).

**Table 1:** Patient characteristics at baseline

Factor	Strata	*n*	%
Total		13	100
Age (years)	Median [range]: 43 [22–68]		
Sex	Male Female	7 6	54 46
Disease extent	Liver Lung Liver + Lung Liver + Lymph nodes/soft tissue Lung + Lymph nodes/soft tissue Liver + Lung + Lymph nodes/soft tissue	3 3 4 1 1 1	23 23 31 8 8 8
Symptomatic disease	Yes No	4 9	31 69
Serosal effusion	Yes No	4 9	31 69
Progressive disease*	Yes No	10 3	77 23

*Progressive disease: Progression according to RECIST or clinical progression

### Treatment and toxicity

The median treatment duration was 8.8 months, with six patients remaining on treatment > 2 years. By the end of follow-up, three patients were still on treatment with PAZ. In seven patients (54%), treatment was discontinued due to tumor progression. Additionally, one patient (8%) stopped treatment due to toxicity (diarrhea, nausea and fatigue), another (8%) due to a new lymphoma diagnosis, and one more because of sustained long-term disease stabilization. Six patients (46%) underwent second-line treatment for EHE: three (23%) with sorafenib and three (23%) with doxorubicin.

**Fig. 3:**
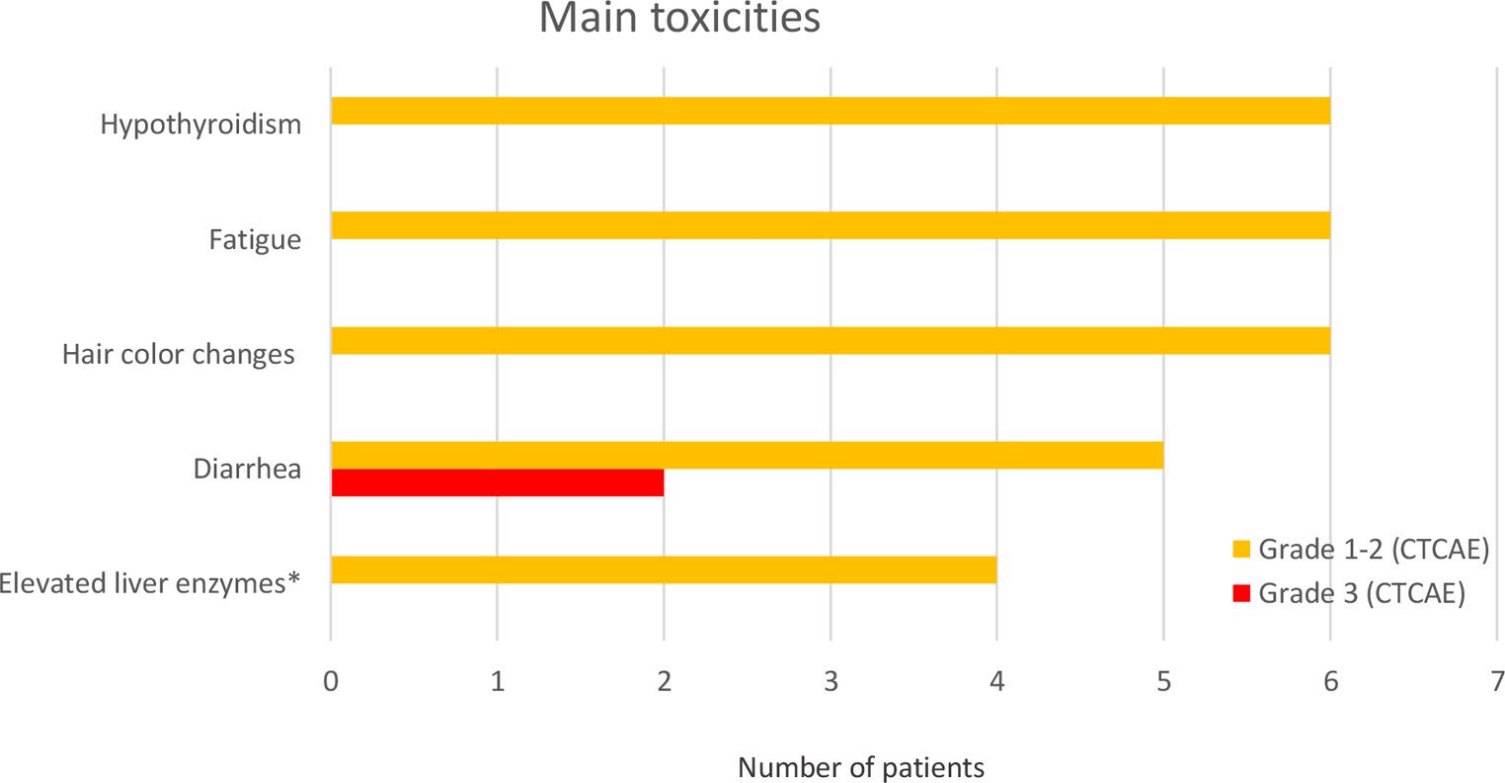
The most common toxicities in patients with epithelioid hemangioendothelioma (EHE) during treatment with pazopanib (PAZ). *Transaminases and/or gamma-glutamyl transferase (gGT) CTCAE: Common Terminology Criteria for Adverse Events

Three patients (23%) required permanent dose reduction of PAZ due to diarrhea (*n* = 2) and the combination of fatigue, myalgia and skin irritations (*n* = 1). In four patients (31%), the therapy was temporarily discontinued due to side effects. No grade 4 or 5 toxicity was reported. Grade 3 toxicity, manifesting as diarrhea, was observed in two patients. The most common toxicities (grade 1–2) were fatigue (*n* = 6, 46%), hypothyroidism (*n* = 6, 46%), hair color changes (*n* = 6, 46%), diarrhea (*n* = 5, 38%), and liver enzyme elevation (*n* = 4, 31%; see Fig. 3). Further reported toxicities were nausea (*n* = 3, 23%), abdominal pain (*n* = 3, 23%), musculoskeletal pain (*n* = 2, 15%) as well as arterial hypertension, peripheral neuropathy, transient peripheral visual disturbances, dysgeusia, and insomnia each in one patient (8%).

### Efficacy

**Fig. 4:**
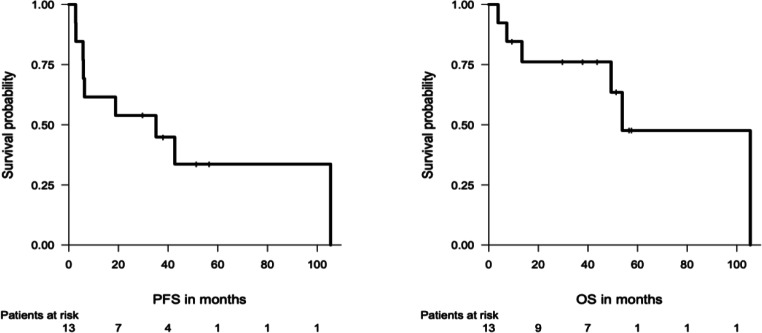
Progression-free survival (PFS) and overall survival (OS) in patients with epithelioid hemangioendothelioma (EHE) after start of pazopanib (PAZ)

At a median follow-up of 51.4 months, the median PFS was 35.1 months. The median OS was 53.8 months, with six deaths (46%) recorded by the end of follow-up (Fig. 4). The 2- and 5-year-OS rates were 76% and 48%, respectively. In patients with evidence of PD according to RECIST or clinical progression at baseline (*n* = 9), the median PFS (according to RECIST) and OS were 12.6 and 105 months, respectively. In patients with serosal effusion/ symptoms at baseline (*n* = 4), the median PFS and OS were 6.1 months and 10.3 months, respectively. In patients with prior progression but without serosal effusions/ symptoms at baseline (*n* = 6), the median PFS and OS were 62.1 and 105 months.

In our cohort, no CR or PR according to RECIST were observed. The best response was SD in eleven patients (85%). The clinical benefit rate (= SD at six months) was 62%. In one patient, clinical progression (pain exacerbation) occurred three months prior to RECIST progression. In two out of four patients with symptomatic disease at baseline (pain and hemoptysis, respectively), treatment with PAZ provided symptomatic relief. One of these patients demonstrated an approximate 80% reduction in ascites with PAZ treatment but did not fulfill the RECIST criteria for PR.

**Fig. 5:**
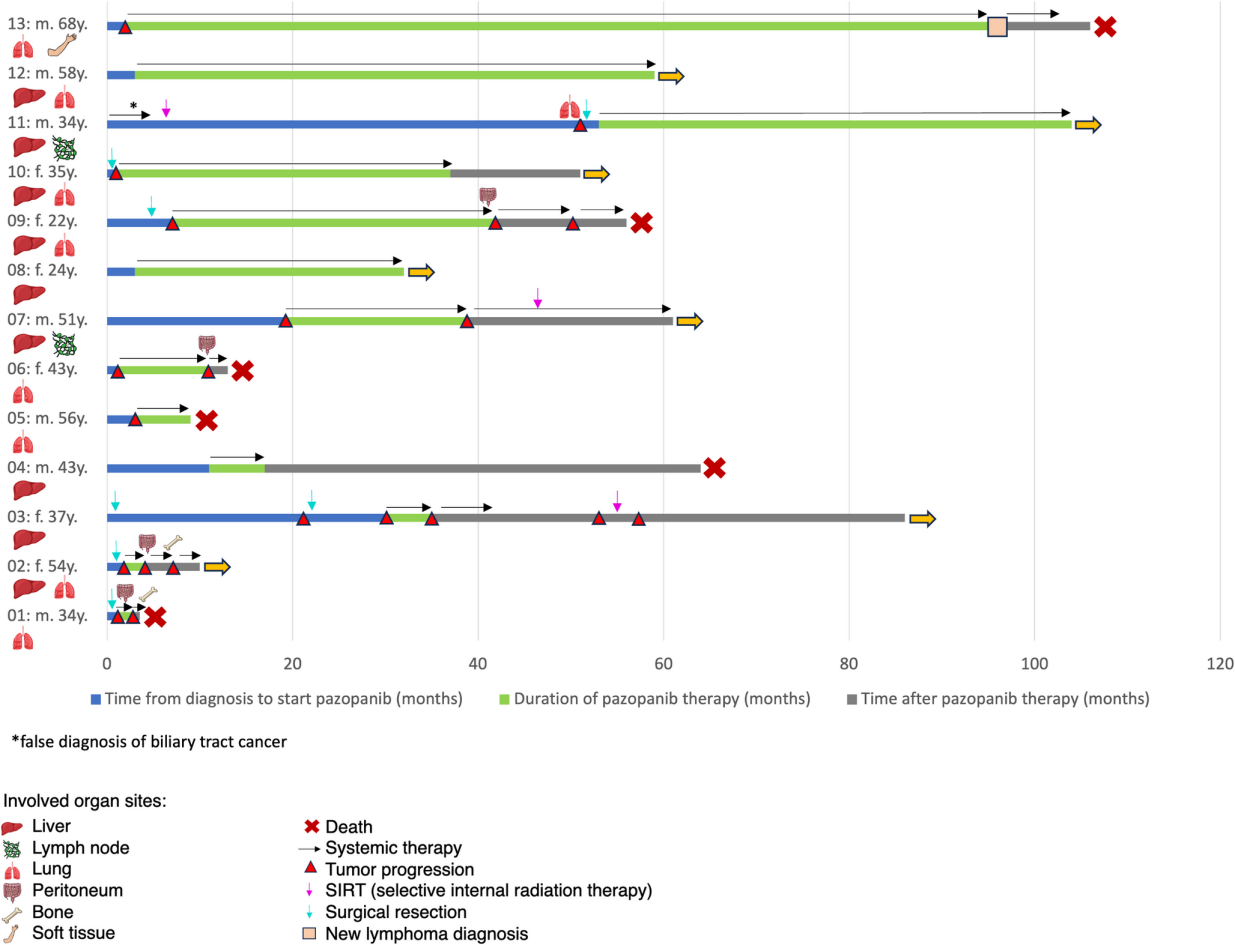
Patients with metastatic epithelioid hemangioendothelioma (EHE) and pazopanib (PAZ) as a first-line treatment

Univariate analysis was performed with regard to clinical prognostic factors (Table 2). There was no significant impact of age, sex, extent of disease, prior tumor progression, and dose reduction/toxicity on PFS and OS. The presence of serosal effusion/symptoms was significantly associated with a worse OS. Figure 5 illustrates the respective courses of disease for each patient.

**Table 2:** Prognostic factors for progression-free survival (PFS) and overall survival (OS), univariate analysis

		PFS		OS	
Factor	Strata	*p*-value	HR (95% CI)	*p*-value	HR (95% CI)
Age	≤ 40 vs. >40	0.71	0.76 (0.18–3.24)	0.90	0.90 (0.15–5.53)
Sex	Female vs. male	0.35	2.05 (0.45–9.21)	0.97	0.96 (0.15–5.98)
Disease extent	Lung vs. Liver	0.84	1.22 (0.17–8.76)	0.34	3.20 (0.29–35.64)
	≥ 2 organs vs. Liver	0.79	0.79 (0.14–4.35)	0.81	1.36 (0.12–15.71)
Progressive disease *	Yes vs. No	0.25	3.46 (0.42–28.64)	0.56	1.96 (0.21–18.18)
Serosal effusion / symptomatic disease	Yes vs. No	*0.062*	4.30 (0.93–19.9)	< 0.001**	5.74e + 09 (0-Inf)
Dose reduction and/or CTCAE ≥ 3	Yes vs. No	0.10	0.26 (0.05–1.33)	0.27	0.29 (0.032–2.64)

*Progressive disease: Progression according to RECIST or clinical progression at baseline

**Due to the small sample size and large HR estimate, the likelihood ratio test was used to indicate significance

CTCAE: Common Terminology Criteria for Adverse Events

## Discussion

This study analyzed a cohort of thirteen patients with metastatic EHE and first-line treatment with PAZ between 2012 and 2023 at our institution. Considering the ultra-rare nature of EHE and the lack of approved active therapies available, reporting clinical outcomes is essential to improve the therapeutic management of this challenging disease.

At a median follow-up of 51.4 months, the median PFS and OS from start of PAZ were 35.1 and 53.8 months, respectively. No responses according to RECIST were observed. Patients with evidence of prior clinical progression or progression according to RECIST (*n* = 10) had a median PFS and OS of 12.6 and 105 months, respectively. Three patients did not show prior tumor progression before treatment start which was an inclusion criterion in the majority of previously published studies on systemic treatments in EHE (Chevreau et al. 2013; Kollár et al. 2017; Stacchiotti et al. 2021b; Schuetze et al. 2024). These patients were directly treated with PAZ to prevent further metastatic spread given their liver-limited disease and the potential option of a curative liver transplant (Lerut et al. 2007). However, one patient declined a liver transplant, another had histological confirmation of metastatic disease shortly after starting PAZ, and another is still undergoing evaluation for transplant.

Kollár et al. reported a median PFS and OS of 26.3 months and an ORR of 20%, with nine out of ten patients having received at least one prior systemic therapy before start of PAZ (Kollár et al. 2017). This study suggests that RECIST responses can be achieved in EHE patients treated with PAZ. In a large retrospective case series from the World Sarcoma Network, twelve patients received PAZ with no responses and a median PFS and OS of 2.9 and 8.5 months, respectively. In this study, seven patients received at least one prior systemic therapy before PAZ (Frezza et al. 2021). However, both studies lacked information about the clinical characteristics and the molecular profile of the included patients.

In the mentioned international experts’ consensus paper, sirolimus is recommended as a first-line treatment based on a retrospective study of 38 patients conducted by Stacchiotti et al.(Stacchiotti et al. 2016, 2021a, b). This study reported a median PFS of 13 months and OS of 18.8 months with an ORR of 11%, including four PR. The patient cohort was predominantly treatment-naïve (81.6%). Available phase II studies on trametinib (*n* = 42), sorafenib (*n* = 15) and bevacizumab (*n* = 7) in patients with advanced EHE found lower survival data compared to our study (median PFS: 10.4, 6.0 and 9.0 months, respectively) but demonstrated partial responses according to RECIST in some patients (ORR: 3.7, 13.3 and 28.6%, respectively) (Agulnik et al. 2013; Chevreau et al. 2013; Schuetze et al. 2024). (Schuetze et al. 2024).

Serosal (pleural/peritoneal) effusion has been identified as an unfavorable prognostic factor in EHE (Rosenbaum et al. 2020; Stacchiotti et al. 2021b). In affected patients, sirolimus had limited activity with a median PFS of 4.8 months compared to 47.8 months in patients without serosal effusion (Stacchiotti et al. 2021b). In our study, the median PFS was 6.1 months in this subgroup of only four patients compared to 62.1 months in patients without serosal effusion but prior progression. However, one patient had a PFS of 35.1 months and benefited in terms of ascites reduction and symptomatic relief. This indicates the efficacy of PAZ in certain patients with serosal effusions and/or symptomatic disease and underscores the need for additional stratification factors.

The recently published SARC33 trial evaluating trametinib in EHE with WWTR1::CAMTA1 fusion did not meet its primary endpoint with an ORR of 3.7% but led to significant improvement of pain intensity and interference (Schuetze et al. 2024). In a commentary on this trial, B. Van Tine and S. Haarberg questioned whether RECIST is an appropriate endpoint in rare tumor trials with unknown biology of response as there might be more relevant outcomes, such as pain improvement. They emphasized that RECIST does not account for non-measurable lesions, such as pleural effusions, which are common in EHE. They suggest placebo-controlled cross-over design trials as the best trial design in ultra-rare tumors with potentially indolent courses of disease (Van Tine and Haarberg 2024). Our findings support this view: while no RECIST responses were observed, two patients with symptomatic disease and serosal effusions experienced marked symptom relief, underscoring the limited utility of RECIST in evaluating treatment responses in EHE.

Sirolimus is currently the preferred systemic treatment option at our institution in accordance with international recommendations. However, our survival rates and the clinical responses might indicate an activity of PAZ similar to sirolimus in EHE. Therefore, the different toxicity profiles of the two treatments should be considered, particularly in the context of a potential long-term therapy. The reported toxicities are in line with previous studies on PAZ in STS (Van Der Graaf et al. 2012; Lee et al. 2019). Besides diarrhea, liver toxicity and fatigue, and less severe common side effects, such as hair color changes, should be discussed before the start of PAZ. Comorbidities and patient preferences should be included as important factors in the treatment decision.

Limitations of our study include the small size of our cohort and the retrospective design, both typical for ultra-rare tumors. Our findings should be interpreted with caution as the observed long-term survival could be influenced by the inherent disease biology rather than predominantly by the effect of PAZ. Furthermore, we included three patients without confirmation of a characteristic gene fusion. This is the first study to report survival outcomes for PAZ as a first-line treatment in patients with metastatic EHE. The long survival outcomes as well as clinical responses and a good tolerability underline the value of PAZ in patients with EHE. PAZ is active and safe in patients with metastatic EHE and can be discussed as an alternative to sirolimus in specific patient subgroups. Additional predictive biological and clinical biomarkers are required to better stratify patients and optimize the treatment of this heterogeneous disease. Furthermore, our findings emphasize the importance of considering alternative response criteria beyond RECIST for evaluating treatment effects in rare tumors.

## Data Availability

No datasets were generated or analysed during the current study.
